# A Knowledge Transfer Approach to Map Long-Term Concentrations of Hyperlocal Air Pollution from Short-Term Mobile Measurements

**DOI:** 10.1021/acs.est.2c05036

**Published:** 2022-09-19

**Authors:** Zhendong Yuan, Jules Kerckhoffs, Gerard Hoek, Roel Vermeulen

**Affiliations:** † Institute for Risk Assessment Sciences, Utrecht University, 3584 CK Utrecht, The Netherlands; ‡ Julius Centre for Health Sciences and Primary Care, University Medical Centre, University of Utrecht, 3584 CK Utrecht, The Netherlands

**Keywords:** mobile monitoring, air pollution mapping, LUR modeling, transfer learning

## Abstract

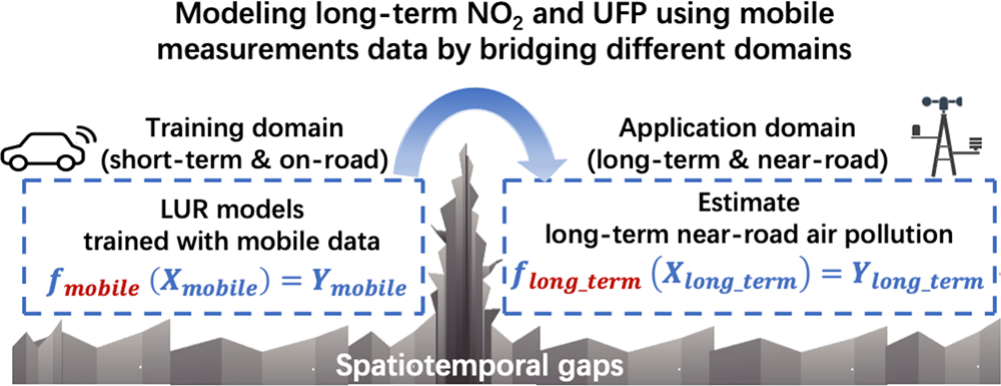

Mobile measurements are increasingly used to develop spatially explicit (hyperlocal) air quality maps using land-use regression (LUR) models. The prevailing design of mobile monitoring campaigns results in the collection of short-term, on-road air pollution measurements during daytime on weekdays. We hypothesize that LUR models trained with such mobile measurements are not optimized for estimating long-term average residential air pollution concentrations. To bridge the knowledge gaps in space (on-road versus near-road) and time (short- versus long-term), we propose transfer-learning techniques to adapt LUR models by transferring the mobile knowledge into long-term near-road knowledge in an end-to-end manner. We trained two transfer-learning LUR models by incorporating mobile measurements of nitrogen dioxide (NO_2_) and ultrafine particles (UFP) collected by Google Street View cars with long-term near-road measurements from regular monitoring networks in Amsterdam. We found that transfer-learning LUR models performed 55.2% better in predicting long-term near-road concentrations than the LUR model trained only with mobile measurements for NO_2_ and 26.9% for UFP, evaluated by normalized mean absolute errors. This improvement in model accuracy suggests that transfer-learning models provide a solution for narrowing the knowledge gaps and can improve the accuracy of mapping long-term near-road air pollution concentrations using short-term on-road mobile monitoring data.

## Abstract 2

The proposed transfer-learning LUR model can map long-term near-road air pollution concentrations in fine spatial resolution by integrating mobile and long-term near-road measurements.

## 1 Introduction

Quantifying chronic health effects of air pollution requires accurate maps of long-term average air pollution at a fine spatial granularity. Mobile monitoring campaigns have shown to be suitable to measure detailed air pollution concentrations on streets. With substantial spatial coverage, mobile measurements are increasingly used to build land-use regression models (LUR) for estimating air pollution concentrations with high spatial resolutions for large spatial areas.^1−9^

Ideally, with multiple repeated measures on roads over a long period of time, the mean of mobile measurements should be able to fully represent the on-road long-term concentrations (e.g., annual average). However, several practical factors introduce additional biases to this representation when attempting at mapping residential air pollution using mobile measurements. Restricted by the length of campaigns and the number of collection vehicles, usually only a few seconds can be measured at each location, especially for large study regions. Chambliss et al.^10^ argued that the temporal scarcity of mobile measurements poses a challenge of representing long-term concentrations. They found only a modest correlation between mobile and nearby long-term measurements in a large mobile monitoring campaign in Oakland. In addition to the temporal difference, since all mobile measurements are on roads, there is also a spatial difference between on-road measurements and near-road concentrations. Kerckhoffs et al. found that nitrogen dioxide (NO_2_) and ultrafine particle (UFP) predictions made by LUR models based on mobile monitoring are approximately 15–30% higher than home-outdoor stationary measurements.^6,7,11^ Moreover, potentially different collection instruments used in mobile campaigns and long-term monitoring networks also contribute to the systematic difference between mobile measurements and the target long-term measurements.^7,11,12^

These spatiotemporal and instrumental differences between mobile training measurements and the target long-term concentrations make that the conventional empirical LUR model contradicts one of the core assumptions of supervised learning methods. Namely, the training and predicting instances must be subject to a similar distribution^13^ (hereafter referred to as the distribution of data instances over the sample space as the domain^14^). If this assumption is violated, the knowledge learned from the training domain (i.e., mobile on-road instances) will differ from the knowledge in the target application domain (i.e., long-term near-road instances). The knowledge here refers to the association between covariates and the response (concentrations). This difference in knowledge between the training and the target domain is defined as the knowledge gaps. Such knowledge gaps result in models based on solely mobile training data not being optimized for prediction accuracy.^14−16^ This problem is widely recognized as domain shifts in computer science research.

We propose one domain adaptation and one boosting-based transfer-learning algorithm to bridge the knowledge gaps between the mobile and the long-term near-road domain in an end-to-end manner. These methods can directly or indirectly incorporate long-term measurements with mobile monitoring data to adapt the training of LUR models. The core idea is to reweight the training goal from minimizing the loss function in the mobile domain into optimizing the loss function in the desired long-term domain and thus efficiently fit machine learning (ML) models with more appropriate parameters.^13,14,17^

This paper describes to what extent the end-to-end transfer-learning LUR model can bridge the knowledge gaps between the mobile on-road domain and the long-term near-road domain in order to boost the accuracy of mapping long-term air pollution near roads. We limited the goal as near-road air pollution because most long-term routine monitoring sites in the study area (Amsterdam) were deployed near roads. We used data from a 10-month mobile monitoring campaign in Amsterdam, where two Google Street View cars continuously measured NO_2_ and UFP.^11^ Two transfer-learning LUR models were compared to the mobile LUR model based on random forest (RF_LUR) and stepwise linear regression (SLR) trained with mobile measurements only. Prediction accuracy was evaluated by external long-term near-road validation data collected by routine monitoring campaigns.

## 2 Data and Methods

### 2.1 Short-Term Mobile Training Data

The mobile measurements used to train the LUR models were collected by two Google Street View (GSV) cars in Amsterdam from 25 May 2019 to 15 March 2020 (stopped due to COVID lockdown policy). Briefly, 1-second measurements of NO_2_ and UFP were collected on weekdays between 08:00 and 22:00 measured by the CAP sensor and the MiniDiSC sensor, respectively. Both sensors show a high correlation compared to the stationary measurements in previous studies.^7,18,19^ A total of 5.9 million measurements for each pollutant were recorded, along with timestamps and geographic coordinates, covering almost all streets of Amsterdam. Mobile measurements of both NO_2_ and UFP were temporally corrected according to one reference site located in a suburban area of Amsterdam, away from traffic sources. Details of the data collection and preprocessing can be found in our previous work.^11^

The road network in Amsterdam was divided into 50 m road segments (*n* = 46,664). The mobile measurement points were snapped to the nearest road segment (n_NO_2_= 41,919, n_UFP = 42,813). The mean value of the snapped measured points was set as the mobile measurements of the corresponding road segment. Drive passes were defined as the number of days that the collection vehicles drove through a road segment. On average, each street segment consisted of 3–10 s measurements per drive-pass and eight unique drive-passes (see the distribution of drive-pass in Appendix Figure S1).

### 2.2 GIS Predictors

The predictors used to predict air pollution concentrations consist mainly of three components: (1) land use from the Copernicus CORINE 2018 dataset,^20^ which is a large pan-European land use database; (2) traffic variables from the national traffic databases in the Netherlands such as traffic counts and road types;^21^ and (3) population density data from the Netherlands Environmental Assessment Agency.^22^ The specific variables including evaluated buffer sizes are summarized in Appendix Table S1.

### 2.3 External Long-Term Measurements

**Figure 1:**
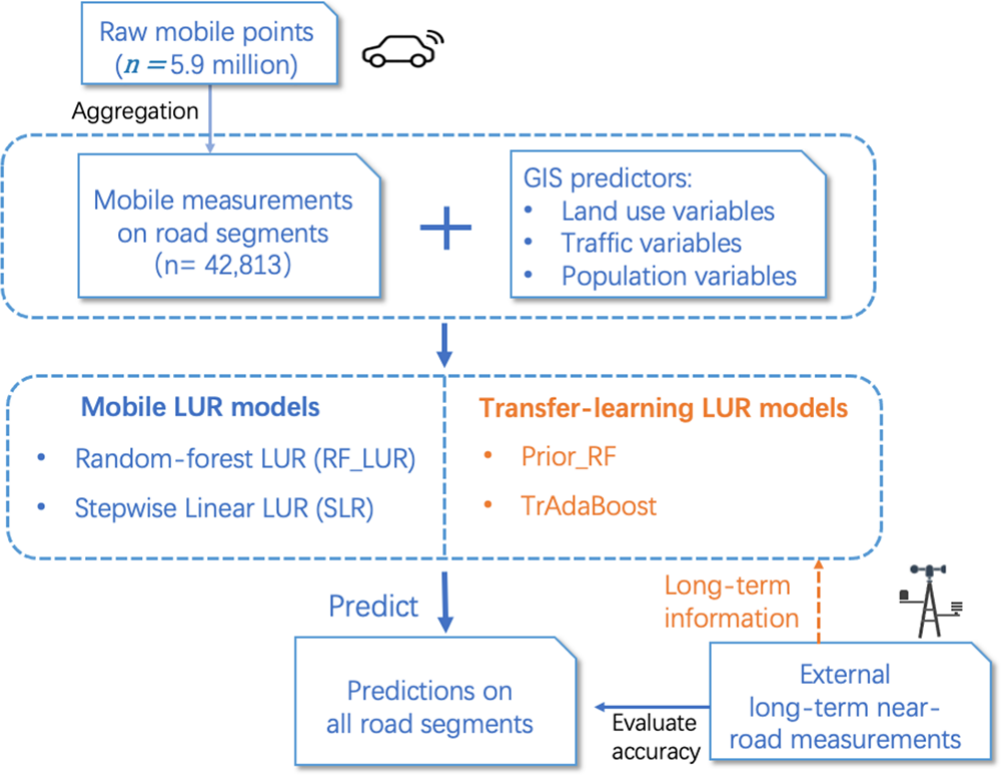
Data and methods involved in developing conventional LUR and transfer-learning LUR models. Two conventional LUR models were implemented as baseline models, namely, stepwise linear LUR model (SLR) and standard random forest LUR model (RF_LUR). Prior_RF and TrAdaBoost are two variants of transfer-learning LUR models that incorporated external long-term information into the training of mobile monitoring data. The accuracy of TrAdaBoost was evaluated using half of the external long-term air pollution measurements. SLR, RF_LUR, and Prior_RF were validated using the full set of external long-term measurements.

Long-term measurements refer to stationary measurements that consecutively measure air pollution at a location for a long period of time. These long-term measurements provide more temporal coverage but at a smaller number of locations. We use the term “near-road” here for measurements near all roads including minor and major roads. We applied these long-term near-road measurements to validate the accuracy of LUR models in terms of predicting long-term near-road concentrations and for the development of the transfer-learning LUR models. For this purpose, long-term Palmes NO_2_ measurements collected by the Dutch Municipal Health Service (GGD) were used.^23^ This data consists of repeated 4-weekly average passive measurements by Palmes tubes located on road-side building façades and lampposts. A total of 82 monitoring sites were located within 30 m of road segments and had complete data during the time period of the mobile monitoring campaign. Their averaged distance to the centerline of the nearest road is about 7 m. For UFP, we used data from the EXPOsOMICS study encompassing 17 sites (on the house façade with the monitor—MiniDiSCs; on average 12 m to the nearest road centerline) measured continuously for repeated 24 h on three different days in different seasons (3 × 24 h) in Amsterdam from 2014 to 2015.^12,24^ The average of the measurements from the 3 days was used. Together with the corresponding environmental predictors, they represent the long-term near-road knowledge. The data flow and models used are summarized in Figure 1.

### 2.4 Model Development

We implemented and compared four LUR models (summarized in Table 1). As a baseline, two mobile LUR models were trained using mobile measurements only and validated on the full set of external long-term data following previous published work.^4,8,10,11^ Next, we implemented two transfer-learning LUR models to mitigate the knowledge gaps between the mobile and the long-term near-road domain by incorporating mobile measurements with information derived from long-term measurements.

**Table 1:** Summary of Models and Comparisons

Models		Algorithms	Training data	Validation data
Mobile LUR model	SLR	Linear regression	Mobile data	Full external long-term data
	RF_LUR	Random forest (RF)	Mobile data	Full external long-term data
Transfer-learning LUR model	Prior_RF	Adapted RF	Mobile data and the ratio of probability distributions between mobile and external long-term measurements	Full external long-term data
	TrAdaBoost	TrAdaBoost.R2	Mobile data and half of the external long-term data	Half of the external long-term data
Sensitivity test				
SLR_half		Llinear regression	Mobile data	Half of the external long-term data
RF_LUR_half		Random forest	Mobile data	Half of the external long-term data
Prior_RF_half		Adapted RF	Mobile data and the ratio of probability distributions between mobile and half of the external long-term measurements	Half of the external long-term data

#### 2.4.1 Transfer-Learning LUR Models

Transfer-learning methods can transfer the knowledge learned from a prior task as a starting point to train a new model on a different but related task, as this requires less training data.^13,26^ In this work, we transferred the prior mobile knowledge extracted from mobile measurements into the long-term domain represented by long-term near-road measurements. We explored two instance-based transfer-learning methods: (1) TrAdaBoost, a boosting-based transfer-learning algorithm,^16^ and (2) Prior_RF, a domain adaptation technique.^14^

TrAdaBoost is implemented as the Two_stage_TraAdaBoost.R2. TraAdaBoost.R2 is an adapted AdaBoost algorithm that is a popular boosting-based ensemble learning algorithm. Ensemble learning is an ML paradigm where multiple models (often called “weak learners)” are trained to solve the same problem and combined to get better results. As an ensemble learning algorithm, boosting methods are designed to train these weak learners sequentially in an adaptative way: each weak learner in the sequence is fitted by giving more weights to instances in the training dataset that caused higher errors by the previous weak learner in the sequence. Intuitively, each weak learner focuses on fitting the most difficult instances in each boosting iteration. At the end, these weak learners are combined to form a “strong” model that is accurate at predicting all the cases learned from the training instances. In our work, TrAdaBoost.R2 directly merges the mobile monitoring data (source instances) (*x*_s_, *y*_s_) with the long-term observations (target instances) (*x*_t_, *y*_t_) to form a single dataset and assign equal initial weights to each instance. These initial weights will be updated during each boosting iteration, according to the absolute errors of predicting target instances. In this way, the source instances that are similar to the target data are emphasized (larger weights) while the different instances are de-emphasized.^13,16^ As an improved version of TrAdaBoost.R2, Two_stage_TraAdaBoost.R2 adjusts instance weights in two stages. In the first stage, at each boosting step, TrAdaBoost decreases the relative weights of source instances that are different from the target instances. In the second stage, the weights of all source instances are frozen while the weights of the target instances that are different from the source instances are increasing.^16^

Prior_RF is a domain adaptation method that reweights the risk function (refers to the expected value of the loss function) of RF by the ratio that reflects the difference between mobile (source) and long-term (target) concentrations. The goal of training a conventional RF model is to identify the parameters and structures of the model for minimizing the risk function in the target domain *R*_t_(*f*) by minimizing the approximated risk function in the source domain *R*_s_(*f*). Therefore, the distributions of the covariates and the response (concentration measurements) in the sample space between training and prediction are required to be similar. When they are different, the domain adaptation algorithms are used to push the *R*_s_(*f*) closer to *R*_t_(*f*) by re-reweighing *R*_s_(*f*) with the ratio of the prior probability distribution between source and target samples ().^14^ RuLSIF (relative unconstrained least-squares importance fitting) is then applied to estimate this ratio directly from the discrete observations of its numerator and denominator.^25,26^ Afterward, the training instances reweighted by this ratio are fed into the training of the conventional RF algorithm. Additionally, in this paper, to better compare the performance between RF_LUR and Prior_RF, the hyperparameters of Prior_RF are kept the same as in RF_LUR, such as number of trees, number of random splitting variables, and maximum of terminal nodes.

#### 2.4.2 Mobile LUR Models

The mobile LUR model refers to the LUR model trained exclusively with mobile measurements. As a linear-regression-based mobile LUR model, the SLR model was implemented following previously described criteria.^11^ The SLR model started with an empty model (intercept only) and then variables were added based on adjusted *R*^2^.^6,7^ Variables were only added when the direction of the association was as predefined, e.g., positive for traffic intensity (see the directions for each covariates in Appendix Table S1).

The RF_LUR model is based on the conventional RF algorithm that is a popular tree-based ML algorithm and has been applied previously in modeling air pollution.^1,4^ To avoid overfitting, the mobile training data were divided into a split of 70% training and 30% test data. RF was trained on the 70% training data based on the fivefold cross-validation. To obtain the best performance, the RF was fine-tuned by the discrete hyperparameter search. The best model was then applied to predict the 30% test data. When the training accuracy is similar to the test accuracy, the model is considered not overfitting.

### 2.5 Model Comparisons and Sensitivity Tests

TrAdaBoost requires directly involving a number of long-term instances as inputs. We input mobile and half of the long-term measurements (random split) to train the TrAdaBoost model (*n*_NO2_train_ = 41; *n*_UFP_train_= 9) and used the other half of the long-term measurements as validation data (*n*_NO2_val_ = 41; *n*_UFP_val_= 8). However, the random splitting may bring additional uncertainty to the stability of models, due to the relatively small size of the validation data especially for UFP. To incorporate this uncertainty, we repeated the random splitting 20 times with different random seeds. For each iteration, TrAdaboost was trained repeatedly.

The other models, i.e., SLR, RF_LUR, and Prior_RF, were evaluated by the full set of long-term data, because of requiring no long-term data directly in the training stage. To account for the model stability, aligned with TrAdaboost, RF_LUR and Prior_RF models were also trained 20 times with bootstrapped mobile measurements. Additionally, to demonstrate the effect of random splitting and to compare the result more directly, we performed a sensitivity analysis by repeatedly training RF_LUR and Prior_RF using the half long-term data used in TrAdaboost (Table 1).

After training, all the abovementioned models were applied to predict concentrations for all 46,664 road segments. The mean of the predicted road segments within 30 m of long-term monitoring stations was compared to the corresponding long-term external measurements (the parts not used in the training stage), for estimating the prediction accuracy. Three common accuracy metrics were used to compare model performance: (1) the normalized mean absolute error (nMAE), which normalizes the standard MAE by the mean of the validation data. This metric is commonly used to indicate the averaged errors in the prediction tasks; (2) the normalized root mean square error (nRMSE), which normalizes RMSE by the mean of validation data; and (3) goodness of fit estimated by squared Pearson correlation (*R*^2^) calculated by the “R2()” function from the R package “Caret.”^27^

## 3 Results and Discussion

### 3.1 Differences between Mobile and Long-Term Measurements

The mobile measurements reflect the levels of on-road, short-term air pollution during the daytime of weekdays. The long-term measurements represent the near-road long-term air pollution concentrations covering all hours of the day and week. The averaged concentrations of NO_2_ and UFP of the mobile measurements (for all 41,919/42,813 street segments) were higher than those of the external long-term validation data (at 17–82 sites, Table 2).

**Table 2:** Summary of Concentrations from Mobile and Long-Term Monitoring Data

Dataset	Source	Number sites	Concentrations	1st Qu.	Mean	3rd Qu./unit
Mobile measurements	Mobile points aggregated to 50-m road segments	41,919	NO_2_	18.6	27.4	32.0 μg/m^3^
		42,813	UFP	11,480	21,901	26,614 particles/cm^3^
Long-term measurements	Palmes^23^	82	NO_2_	20.9	26.1	30.5 μg/m^3^
	EXPOsOMIC^24^	17	UFP	15,367	18,584	21,419 particles/cm^3^

**Figure 2:**
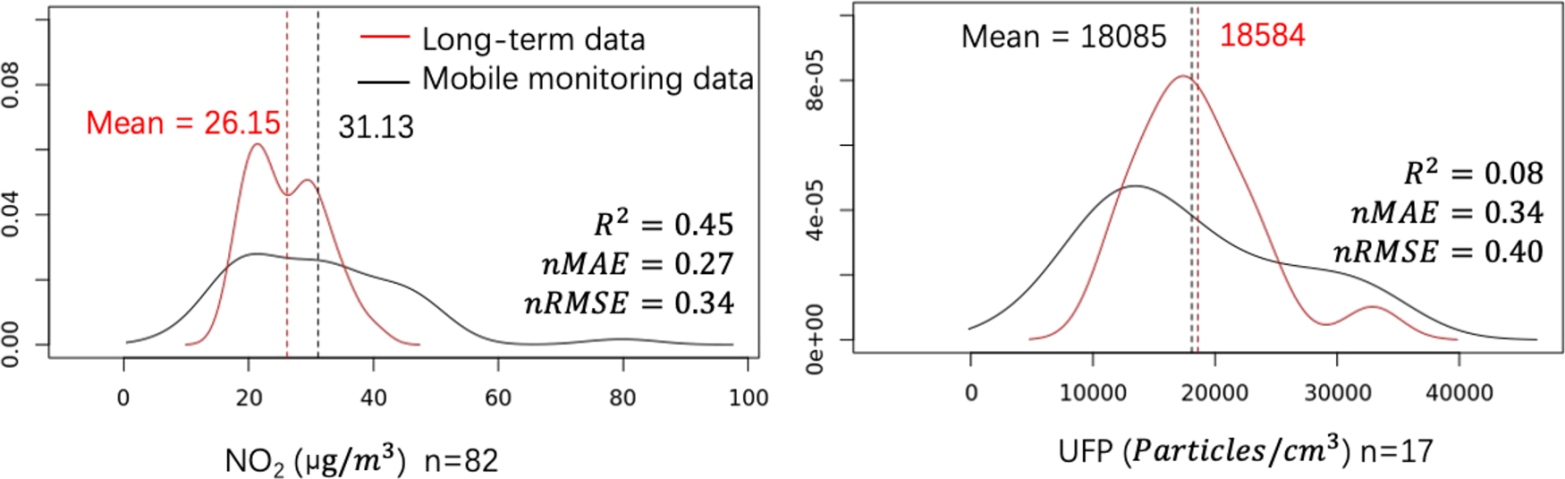
Differences in density distributions between mobile and long-term measurements for NO_2_ and UFP at long-term validation sites. The mean values were marked.

A weak to moderate correlation was observed for UFP (*R*^2^ = 0.08) and NO_2_ (*R*^2^ = 0.45) when comparing the long-term with mobile measurements within 30 m of the external long-term sites. The NO_2_ value of mobile measurements distributes on a wider range as compared to the long-term measurements when plotting the density distribution of measurements at the locations where both mobile and long-term measurements were available (Figure 2). Although the mobile and long-term measurements of UFP distribute in a similar range, the accuracy metrics show they are in a lower correlation than that of NO_2_.

### 3.2 Model Performance

#### 3.2.1 Overall Performance

The performance of all models tested is summarized in Table 3. In general, transfer-learning LUR models were more accurate at estimating long-term near-road air pollution concentrations than conventional LUR models trained on mobile data only. The accuracy improvement in percentage was quantified as shown in Table 4 and calculated using (median_of_transfer_learning – median_of_conventional)/median_of_conventional. TrAdaBoost improved performance by 55.2% and 31.6% over RF_LUR and SLR, evaluated by nMAE for NO_2_. As for UFP, the accuracy gains of Prior_RF were greater in *R*^2^, which were 40% and 86.7% as compared to RF_LUR and SLR models.

**Table 3:** Model Performance of Predicting Long-Term Air Pollution Validated by External Long-Term Data (Mean and 95% CI)

	NO_2_			UFP		
Models	nMAE	nRMSE	*R*^2^	nMAE	nRMSE	*R*^2^
SLR	0.19	0.23	0.49	0.22	0.27	0.20
RF_LUR	0.29 (0.29,0.30)	0.38 (0.38,0.39)	0.53 (0.52,0.54)	0.26 (0.25, 0.27)	0.35 (0.35,0.37)	0.15 (0.13,0.16)
Prior_RF	0.24 (0.22,0.25)	0.31 (0.29,0.32)	**0.62 (0.61,0.63)**	**0.19 (0.19,0.20)**	**0.25 (0.24,0.26)**	**0.28 (0.23,0.33)**
TrAdaBoost	**0.13 (0.11,0.15)**	**0.18 (0.16,0.21)**	0.54 (0.47,0.60)	0.21 (0.18,0.23)	**0.25 (0.23-0.29)**	0.25 (0.18,0.31)
Sensitivity test (mean and 95% CI)						
RF_LUR_half	0.28 (0.27-, 0.30)	0.38 (0.35-, 0.40)	0.54 (0.49, 0.60)	0.26 (0.22,0.30)	0.35 (0.3, 0.40)	0.23 (0.12, 0.34)
Prior_RF_half	0.24 (0.22,0.26)	0.31 (0.29-, 0.33)	0.64 (0.60, 0.68)	0.18 (0.16, 0.20)	0.22 (0.18, 0.26)	0.29 (0.14, 0.43)

**Table 4:** Improvement in Percentage of Transfer-Learning LUR Models Compared to Conventional Mobile LUR Models^a^

	SLR			RF_LUR		
NO_2_ models	nMAE	nRMSE	*R*^2^	nMAE	nRMSE	*R*^2^
TrAdaBoost	–31.6%	–21.7%	+10.2%	–55.2%	–52.6%	+1.9%
Prior_RF	+26.3%	+34.8%	+26.5%	–17.2%	–18.4%	+17.0%
UFP models						
TrAdaBoost	–4.6%	–7.4%	+25.0%	–19.2%	–28.6%	+66.7%
Prior_RF	–13.6%	–7.4%	+40.0%	–26.9%	–28.6%	+86.7%

a Improvement in percentage is calculated using (median_of_transfer_learning – median_of_conventional)/median_of_conventional.

The performance of TrAdaBoost fluctuated more than that of Prior_RF for both NO_2_ and UFP over the 20 iterations (wider 95CI range in Table 3). Part of the fluctuations can be explained by the fact that only half of the long-term measurements were used to calculate the performance of TrAdaBoost, as the 95CI of both Prior_RF_half and RF_LUR_half (validated on 50% of long-term sites) were also larger than those of Prior_RF and RF_LUR (validated on all sites) in the sensitivity test.

RF_LUR was less accurate than SLR for both NO_2_ and UFP, with the only exception of the *R*^2^ for NO_2_ (Table 3). The training accuracy of RF_LUR in terms of *R*^2^ was 0.75 evaluated by the 70% mobile training data for NO_2_ (training *R*^2^ = 0.54 for UFP). The test accuracy was similar to the training accuracy (testing *R*^2^ = 0.73 for NO_2_, *R*^2^ = 0.53 for UFP, evaluated by the other 30% mobile test data).

#### 3.2.2 Density Plot of Predictions

**Figure 3:**
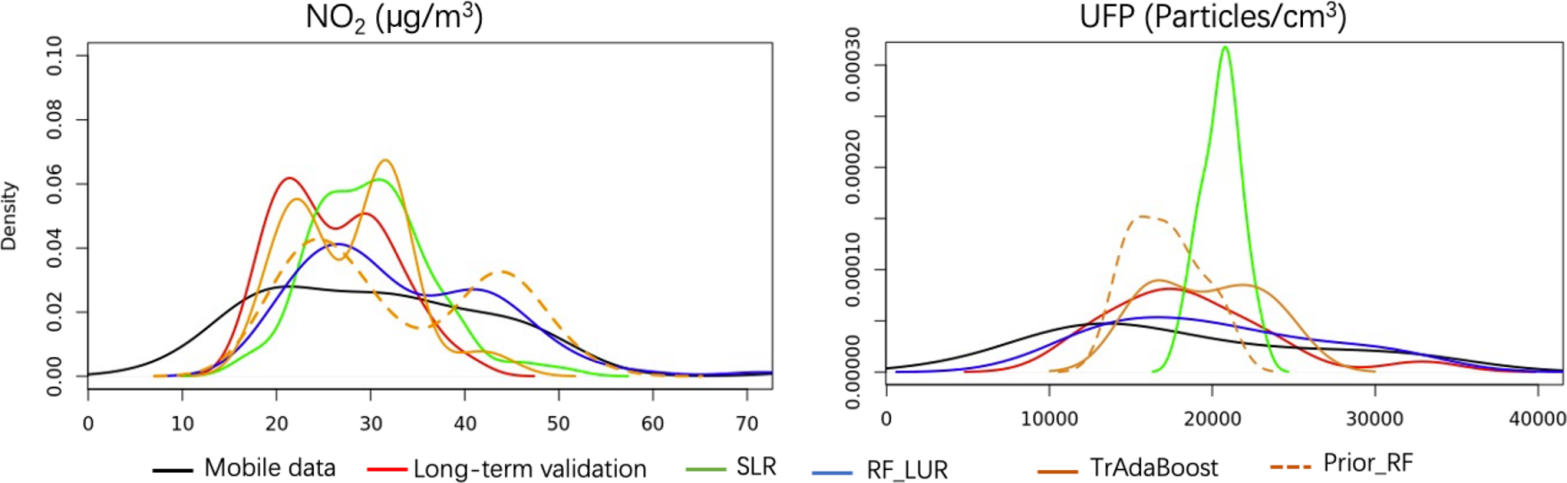
Density plot of predictions and measured long-term concentrations at validation sites. For each method, a model was selected whose performance was the median of the repeated cross-validation performance.

The visual evaluation of the density plots shows that SLR predictions were generally in a narrower range than the desired long-term range, especially for UFP (Figure 3). The predictions of RF_LUR were similarly distributed to the mobile measurements that served as training data but wider than the measured long-term data. In contrast, the predictions of both transfer-learning LUR models were closer to the long-term validation observations than the SLR and RF_LUR models.

#### 3.2.3 Variable Importance and Spatial Distribution Patterns

**Figure 4:**
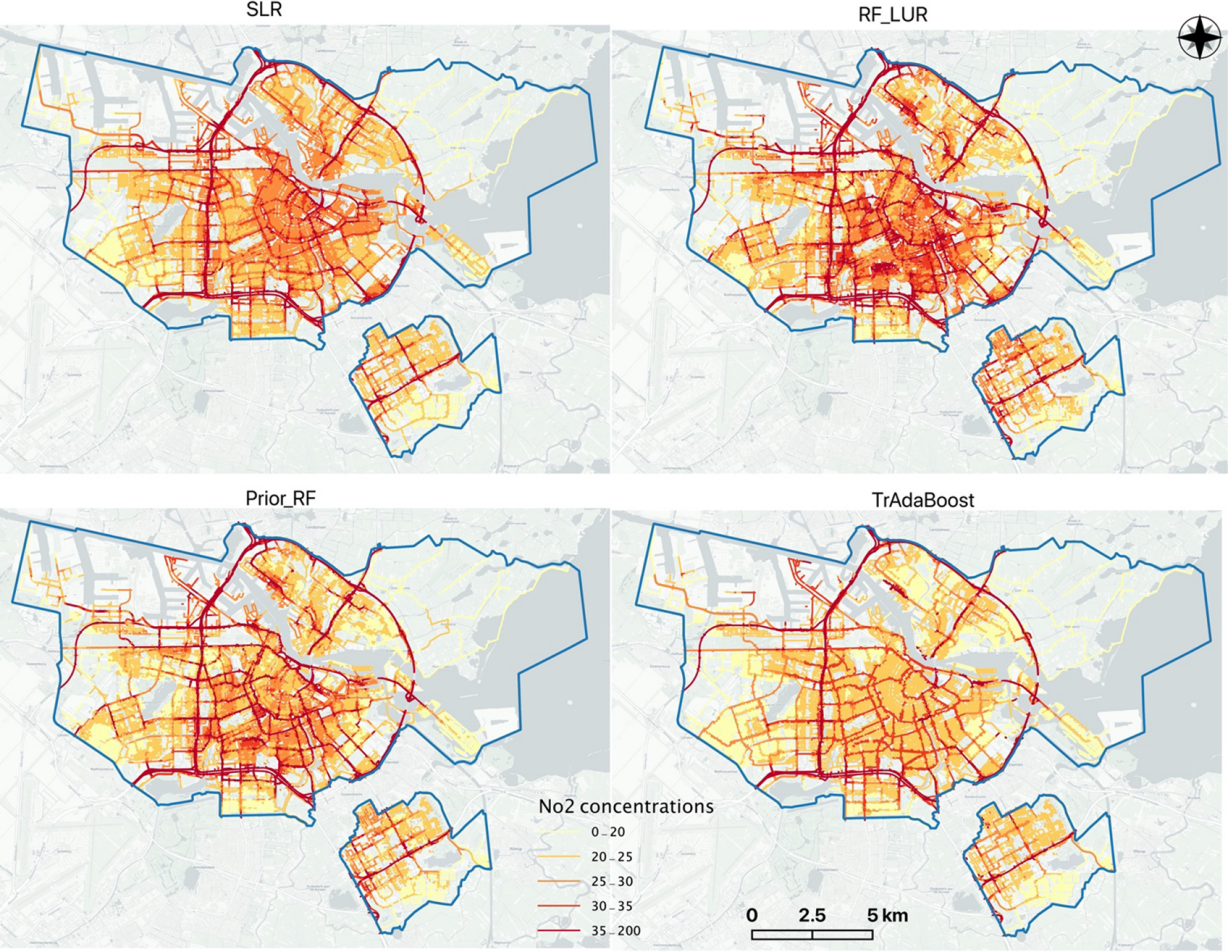
Map of predicted long-term NO_2_ concentration (μg/m^3^) based on various GIS predictors. SLR is one of the conventional linear LUR model. RF_LUR is one of the traditional ML-based LUR models. Prior_RF and TrAdaBoost are two transfer-learning based LUR models that integrate long-term observations with mobile measurements in the training phase.

The selected predictors and corresponding coefficients of the SLR model are presented in Appendix Table S2. Traffic variables were the most important predictors, followed by population and port-related features. Similarly, traffic-related variables were also the main and most important variables for RF_LUR and both transfer-learning models (see Appendix Figure S2). Transfer-learning LUR models considered additionally the population and other environmental contextual information such as the water area and the urban green space. All NO_2_ and UFP maps show that the ring road of Amsterdam is the most polluted area as compared to other locations (Figure 4 and Figure S3 in Appendix).

**Figure 5:**
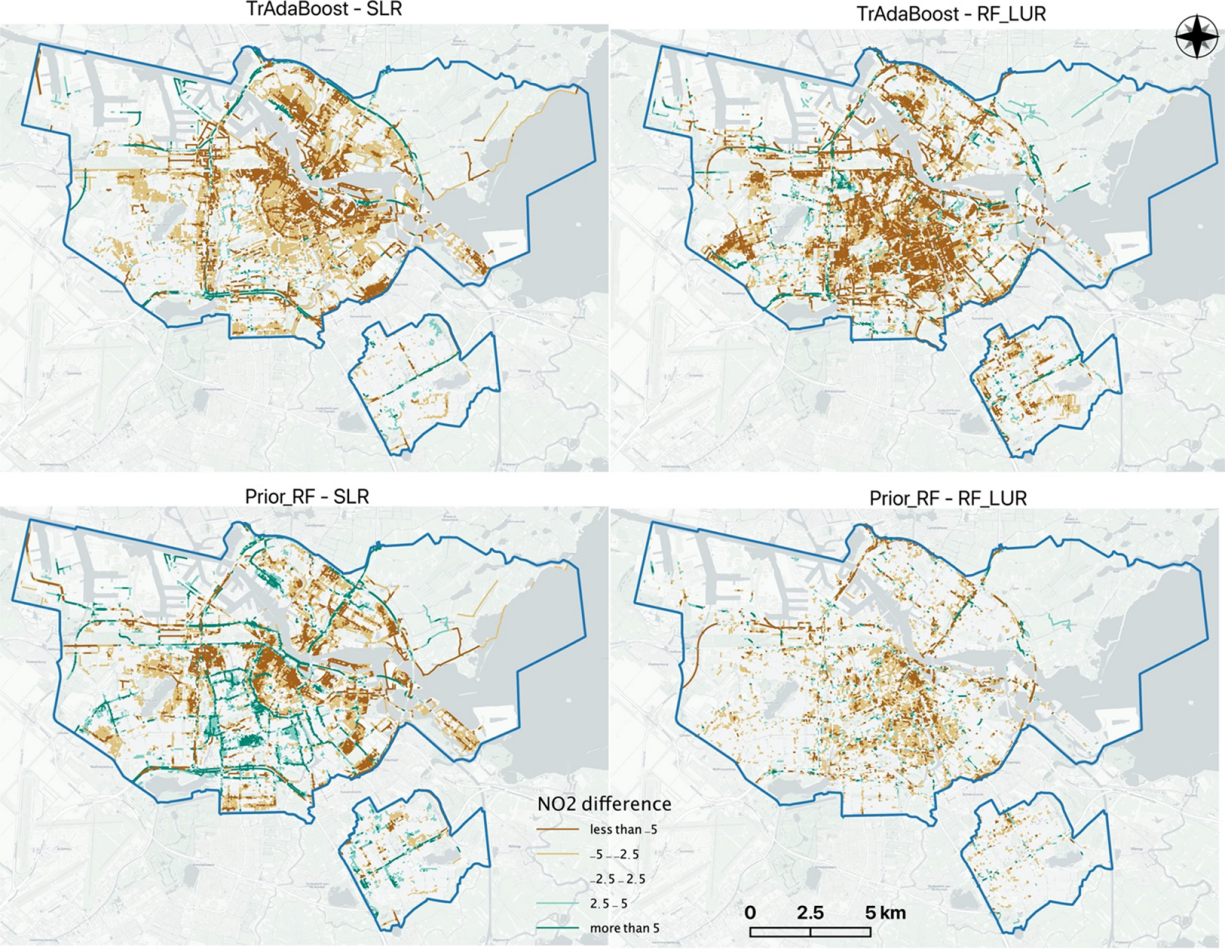
Spatial differences in NO_2_ predictions (μg/m^3^) between transfer-learning LUR and mobile LUR models.

Comparing the spatial differences among the tested models, Prior_RF and TrAdaBoost generally predicted lower NO_2_ concentrations than SLR and RF_LUR at residential locations, especially in the city center (Figure 5). In contrast, at major road locations, TrAdaBoost and Prior_RF predicted higher NO_2_ concentrations than SLR and similar levels to RF_LUR. For UFP, Prior_RF predicted lower concentrations than SLR and RF_LUR for most locations. TrAdaBoost predicted higher UFP concentrations than SLR on the ring road and in the city center than RF_LUR (Appendix Figure S4).

### 3.3 The Issue of Knowledge Gaps in Conventional Mobile LUR Models

When applying mobile measurements to estimate long-term air pollution concentrations near roads, empirical mobile LUR models are hampered by the spatiotemporal and instrumental differences between the training and the application domains. First, mobile measurements often consist of just a few seconds of observations per road segment. In our GSV campaign, two cars collected daytime air pollution on weekdays for 10 months and measured on average 8 drive-passes for each 50 m road segment. In contrast, long-term measurements measure air pollution over a longer period including all hours and days during the study period. Second, in our mobile monitoring campaign, the on-road air pollution is measured. However, significant differences in (traffic-related) air pollution concentrations by the spatial distance to the road have been reported.^11^ In addition, different sensors used in mobile campaigns and regular monitoring networks often bring a certain number of extra differences, although calibrations and collocations are performed.

All of these knowledge gaps originating from space, time, and instruments are at odds with the core assumption of supervised learning methods. Consequently, trained exclusively with mobile measurements, the training accuracy of mobile LUR models (estimated by mobile measurements) is often not equal to their performance in predicting long-term near-road air pollution when validated by long-term near-road measurements.^4,28^ In our study, RF_LUR showed a decrease from its training accuracy (cross-validation based on mobile measurements; *R*^2^ = 0.75 for NO_2_; *R*^2^ = 0.54 for UFP) to the application accuracy (validated by the long-term measurements; *R*^2^ = 0.53 for NO_2_; *R*^2^ = 0.15 for UFP). This is not an overfitting issue, since the training accuracy was similar to the test accuracy when only mobile data were used (*R*^2^ = 0.73 for NO_2_; *R*^2^ = 0.53 for UFP, evaluated by the other 30% mobile test data). The similar accuracy between the training and the test mobile data indicates that the model learned from the mobile instances generalized well to other datasets in the same domain (mobile domain).When the application domain shifts into another domain (e.g., the long-term domain), the predictions accuracy will not necessarily be equal to the training accuracy.

Both the RF_LUR model and SLR face the same issues, as both are supervised learning algorithms trained solely with mobile measurements. However, RF_LUR was less accurate than SLR (Table 4). This suggests that ML-based LUR models tend to be more impacted by the knowledge gaps than linear-LUR models. Several recent mobile studies also found no significant improvement of ML and even, in some cases, worsening of model performance in mapping long-term concentrations compared to linear regressions.^4,28,29^ ML models consist of a more complex structure with a larger number of parameters that need to be optimized based on the training samples. The mobile training data made the model fully delineate the mobile knowledge. However, this mobile knowledge did not translate to the long-term domain. Consequently, the advantage of ML, namely, accurate fitting, turns out to be the major limitation when the prediction domain shifts away from the training domain.

### 3.4 Transfer-Learning LUR Models Can Narrow the Knowledge Gap

Despite the complex spatiotemporal and instrumental differences between the mobile and long-term concentrations, with a limited amount of target (i.e., long-term) information, our proposed transfer-learning LUR models were able to narrow the knowledge gap by transferring learnings from the mobile domain into the long-term domain in an end-to-end paradigm. This end-to-end paradigm was implemented by assigning smaller weights to the mobile training instances that were different from the target long-term near-road instances and emphasizing the target instances that are different from mobile instances to adjust the risk function of LUR models. This pushed the LUR model to learn more from the long-term near-road instances while still optimally utilizing the mobile instances to capture the detailed hyperlocal variations at the same time. Compared to mobile LUR models trained with mobile instances only, transfer-learning LUR models achieved smaller errors in predicting long-term near-road air pollution concentrations (Tables 3 and 4). This comparison is more straightforward when comparing Prior_RF and RF_LUR models, since they are both based on the RF algorithm with the same hyperparameters. The only difference is with and without the adaptation of long-term near-road information.

The mobile measurements were higher than the long-term near-road measurements (Table 2), due to the on-road measurements during the daytime of weekdays (generally busier than other timeslots) as well as more repeats on major roads. This resulted in overestimations of air pollution by mobile LUR models, especially on residential roads. The lower estimations of NO_2_ and UFP from TrAdaBoost and Prior_RF predictions suggest that transfer-learning LUR models can correct this biased trend (see the prediction differences in Figure 5 and Figure S4 in Appendix).

Although the performance of TrAdaBoost was validated on a half of the long-term measurements, it is still reasonable to be compared with other LUR models that were validated on the full dataset. In the sensitivity test, validated on only half of the long-term data, the averaged performances of Prior_RF_half and RF_LUR_half were similar to the mean of Prior_RF and RF_LUR for both air pollutants. These similar mean performances estimated on the full and half versions indicate that the mean value of the separated half validation data can represent the full validation data. Thus, the averaged performance of TrAdaBoost can be directly compared to the models validated by the full set of validation data.

### 3.5 The Comparison of Transfer-Learning LUR Models

The performance of transfer-learning models was found to be limited by the number of long-term measurements that can be included in the training phase. Prior_RF is less sensitive to the number of long-term data than TrAdaBoost. In more data-rich situations such as NO_2_, 41 long-term measurements could be used in training to approximate the long-term knowledge. This makes TrAdaBoost stable and accurate to transfer the learned mobile knowledge toward long-term knowledge. In our result, TrAdaBoost achieved better nMAE and nRMSE than Prior_RF for NO_2_ (see Figure 3). In contrast, only nine long-term instances were included in the training of TrAdaBoost for UFP. Given the size of Amsterdam, the low number of long-term instances makes it challenging to approximate the long-term knowledge from these nine instances for TrAdaBoost. In contrast, Prior_RF is based on the ratio of the probability distribution between the mobile and the long-term domains and thus all long-term data could be used to estimate this ratio (n = 17). Therefore, it is less affected by a limited number of long-term instances. With few UFP long-term measurements, Prior_RF achieved better nMAE and nRMSE than TrAdaBoost (see Table 3).

Prior_RF can generally obtain a better *R*^2^ than TrAdaBoost. This may be due to their different strategies of determining weights. TrAdaBoost is designed to adjust the weights of individual instances based on their similarities (defined by the absolute error) to the long-term instances. Thus, TrAdaBoost can better transfer the mobile knowledge toward long-term knowledge in terms of absolute errors. In contrast, Prior_RF reweights the risk function by the ratio of the probability distribution between mobile and long-term measurements. In this way, Prior_RF predictions can obtain a better correlation with the long-term data, which is reflected in the higher *R*^2^ observed for Prior_RF as compared to the TrAdaBoost model (Table 3).

### 3.6 Strengths and Limitations

Although transfer-learning LUR models outperform the mobile LUR methods, a certain level of long-term knowledge in the study area is required. An advantage of selecting Amsterdam as the study area is the large number of long-term NO_2_ regulatory monitoring sites (*n* = 82). In contrast, the number of the long-term UFP measurements used is relatively small (*n* = 17) and could not temporally cover the entire study period. The number and quality of long-term observations directly influence the performance of transfer-learning LUR models. However, at this point, it is not clear how many long-term measurements are sufficient. It will depend on various factors, such as the choice of hyperparameters, the feature space, and the size of study area. According to the study described in this work, it seems that Prior_RF has higher robustness to the number of long-term data than TrAdaBoost. Transfer learning methods in this work focus on modeling long-term average concentrations. Future work could evaluate transfer learning methods for shorter-term exposures (e.g., modeling hourly concentrations).

Our Google Street View campaign extensively measured air pollution at a spatially fine granularity. Together with the external long-term monitoring data, Amsterdam provides a unique opportunity to evaluate empirical LUR methods on the ability of estimating long-term average air pollution concentrations using short-term on-road mobile measurements. Although our collection cars have traveled around Amsterdam for about 10 months and collected air pollution at 1.7 million locations (an average of 8 drive-passes per road segment), such an intensive mobile dataset still lacks temporal coverage for each location and inherently only measures on-road air pollution as compared to the desired long-term concentrations. We emphasized that the spatiotemporal and instrumental differences cause the mobile knowledge learned by empirical mobile LUR models to deviate from the long-term near-road knowledge. By augmenting the mobile data with temporally rigorous long-term and near-road measurements, our proposed transfer-learning LUR methods showed promising ability to narrow these knowledge gaps. These spatiotemporal and instrumental differences likely exist in most long-term air pollution mapping work when using mobile monitoring data. More attention to these knowledge gaps is needed when applying empirical LUR models to map long-term residential air pollution with mobile monitoring data.

## Supporting Information Available

The figures showing the histogram of drive-passes per road segments, the variable importance plot of the tested models, the spatial UFP map, and the UFP spatial difference map and the tables recording the GIS predictor variables. Coefficients of SLR model (PDF)
