# Supplementary material for: A Knowledge Transfer Approach to Map Long-Term Concentrations of Hyperlocal Air Pollution from Short-Term Mobile Measurements
Source: Environ Sci Technol. 2022 Sep 19;56(19):13820–8. doi: 10.1021/acs.est.2c05036 (PMC9535937; doi:10.1021/acs.est.2c05036)
Supplement: Supplementary file 1 — es2c05036_si_001.pdf [file es2c05036_si_001.pdf]

Supplementary Information for:

A knowledge transfer approach to map long-term concentrations of hyperlocal air pollution from short-term mobile measurements

Zhendong Yuan [1,], Jules Kerckhoffs [1], Gerard Hoek[1], Roel Vermeulen[1,2]*

[1]Institute for Risk Assessment Sciences, Utrecht University, 3584 CK Utrecht, The Netherlands

[2]Julius Centre for Health Sciences and Primary Care, University Medical Centre, University of Utrecht, 3584 CK Utrecht, The Netherlands

*Corresponding author

z.yuan@uu.nl

Figure S1: Histogram of drive-passes per road segment calculated from the Google mobile monitoring data.

Figure S2: Top-10 variable importance of the tested ML models for NO_2 and UFP.

Figure S3: Maps of predicted long-term UFP concentrations (particles/cm^3).

Figure S4. Spatial differences in UFP predictions (Particles/cm^3) between transfer-learning LUR and mobile LUR models.

Table S1: Spatial predictor variables with units, a priori defined directions of effect, and buffer sizes in AMS.

Table S2: Coefficients of SLR.

Figure S1. Histogram of drive-passes per road segment calculated from the Google mobile monitoring data.

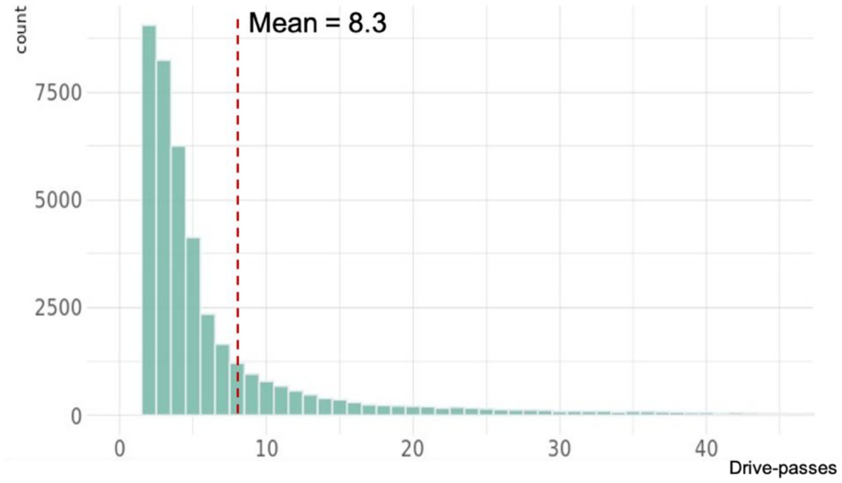

Figure S2: Top-10 variable importance of the tested ML models for NO_2 and UFP.

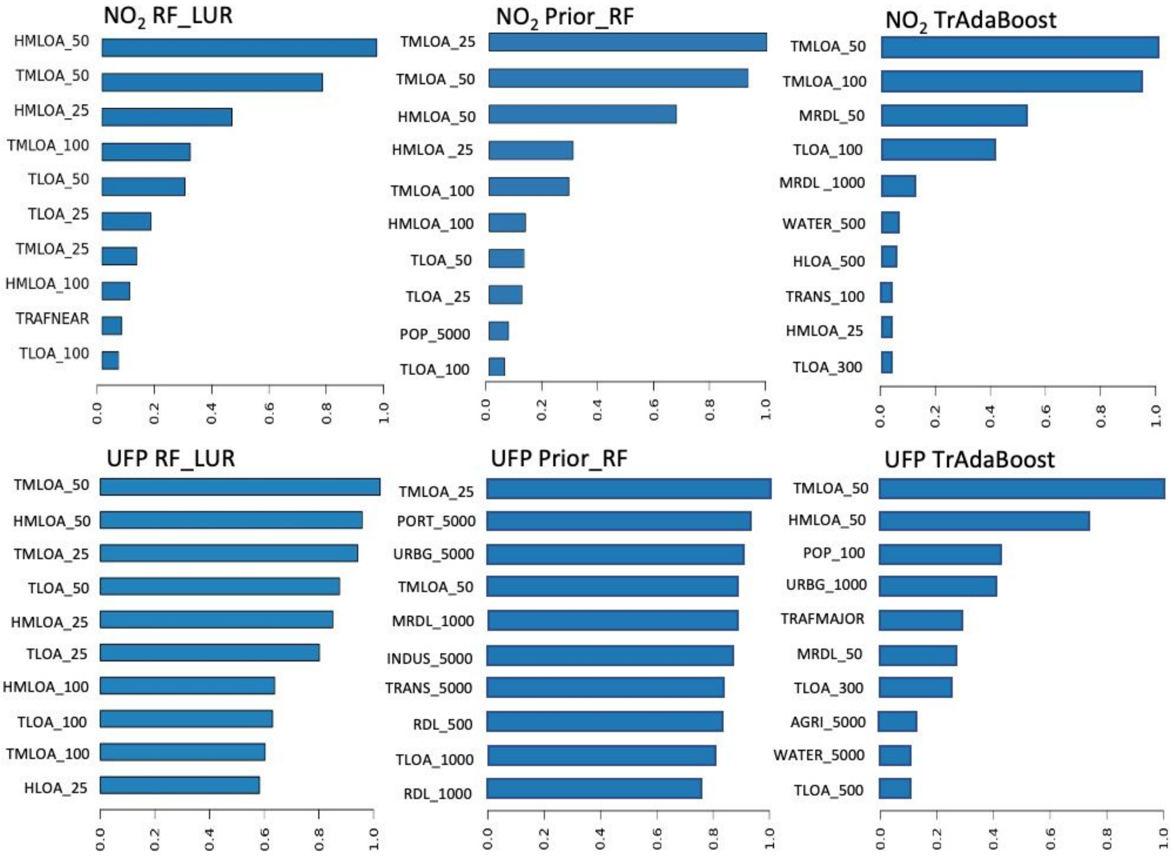

*The full names of features are explained in the Appendix Table S1.

Figure S3: Maps of predicted long-term UFP concentrations(particles/cm^3).

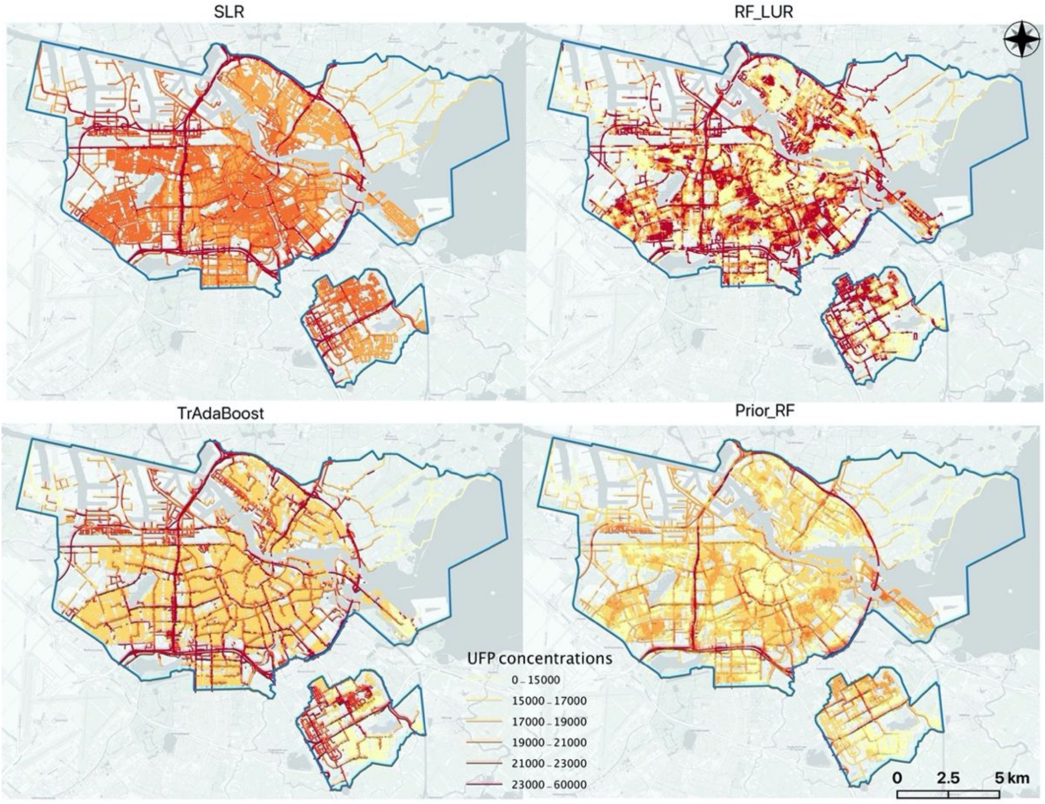

Figure S4. Spatial differences in UFP predictions (Particles/cm^3) between transfer-learning LUR and mobile LUR models.

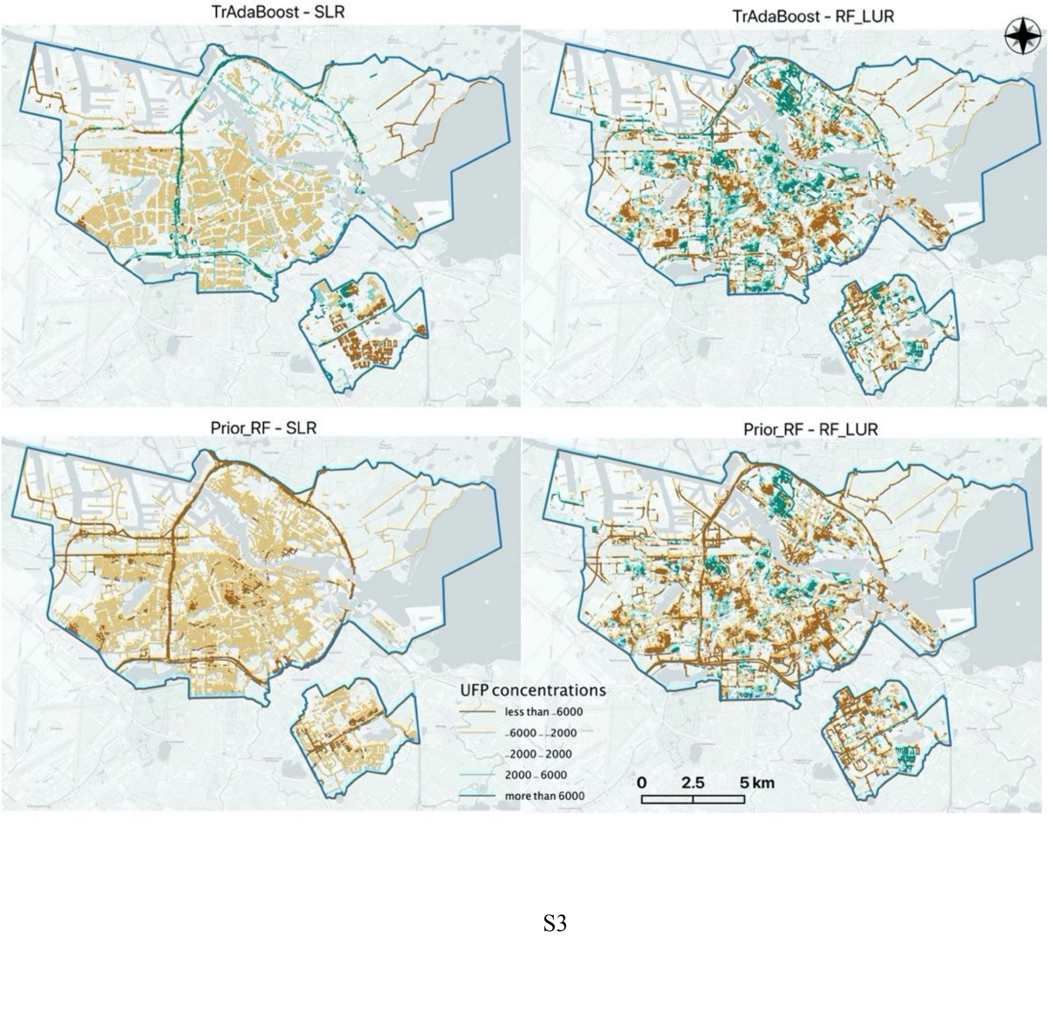

Table S1: Spatial predictor variables with units, a priori defined directions of effect, and buffer sizes in AMS.

Predictor variable	Abbreviation	Units	Direction of effect	Buffer	10th percentile	Mean	90th Percentile
Agricultural land area[1]	AGRI_	m^2	-	100	0	1159	0
				300	0	11288	0
				500	0	34779	64052
				1000	0	178942	649727
				5000	1710991	13290916	30287613
Airport area[1]	AIR_	m^2	+	5000	0	640794	1628347
Industry area[1]	INDUS_	m^2	+	100	0	2006	0
				300	0	17769	41347
				500	0	49074	180131
				1000	0	199831	730248
				5000	2902185	5182292	8091464
Natural and forested areas[1]	NATUR_	m^2	-	100	0	0	0
				300	0	0	0
				500	0	0	0
				1000	0	0	0
				5000	0	429332	2388024
Port area[1]	PORT_	m^2	+	100	0	2648	2516
				300	0	22447	85979
				500	0	59770	258949
				1000	0	226393	956817
				5000	0	6167718	13267782
Residential land area[1]	RES_	m^2	+	100	0	21152	31416
				300	0	183950	282742
				500	0	492795	785396
				1000	104805	1826998	3040804
				5000	17043122	32157817	46811645
Transportation area[1]	TRANS_	m^2	+	100	0	0	0
				300	0	0	0
				500	0	13457	2874
				1000	0	50061	233924
				5000	519010	1340506	2093995
Urban Green area[1]	URBG_	m^2	-	100	0	2351	7952
				300	0	25250	102385
				500	0	79234	268822
				1000	0	361378	852803
				5000	6248972	9362179	13883066
Water[1]	WATER_	m^2		100	0	0	0
				300	0	10238	30716
				500	0	37897	152537
				1000	0	222478	741893
				5000	2904140	8362718	12310634
Population density[2]	POP_	n	+	100	0	319	770
				300	0	2385	5355
				500	25	6091	13410
				1000	1485	21762	46115
				5000	146514	366674	580006
Traffic intensity on nearest road[3]	TRAFNEAR	Veh/day	+		137	9986	22487
Traffic intensity on nearest major road[3]	TRAFMAJOR	Veh/day	+		3115	14212	28042
Heavy-duty traffic intensity on nearest road[3]	HTRAFNEAR	Veh/day	+		0	619	1333
Heavy-duty traffic intensity	HTRAFMAJOR	Veh/day	+		54	980	2138

**on nearest major road[3]**							
**Road length of all roads[3]**	RDL_	m	+	**25**	39	66	100
				**50**	100	187	294
				**100**	254	636	966
				**300**	2029	4880	7083
				**500**	7893	13761	19090
				**1000**	29092	50344	68095
**Road length of all major roads[3]**	MRDL_	m	+	**25**	0	24	91
				**50**	0	69	198
				**100**	0	210	534
				**300**	0	1532	3554
				**500**	0	3787	8800
				**1000**	3015	13627	31882
**Traffic intensity on all roads (sum of (traffic intensity * length of all segments)) [3]**	TLOA_	(Veh/day)*m	+	**25**	0	634722	1579945
				**50**	6393	1815133	4797110
				**100**	78159	5494027	14290643
				**300**	3038453	36918277	1.01E+08
				**500**	13741938	44180744	87625101
				**1000**	62971674	166892417	324383228
**Traffic intensity on all major roads (sum of (traffic intensity* length of all segments)) [3]**	TMLOA_	(Veh/day)*m	+	**25**	0	543194	1387293
				**50**	0	1548534	4473085
				**100**	0	4550786	13415227
				**300**	0	12783795	26453420
				**500**	0	30877644	64537936
				**1000**	26948221	116664854	238567679
**Heavy-duty traffic intensity on all roads (sum of (heavy-duty traffic intensity* length of all segments)) [3]**	HLOA_	(Veh/day)*m	+	**25**	0	40889	59383
				**50**	0	115926	205867
				**100**	1607	336918	909792
				**300**	84212	2085219	8507687
				**500**	387896	2664654	6027990
				**1000**	2550331	10515869	25699398
**Heavy-duty traffic intensity on major roads (sum of (heavy-duty traffic intensity*length of all segments) [3]**	HMLOA_	(Veh/day)*m	+	**25**	0	35363	41295
				**50**	0	100488	141074
				**100**	0	286355	742416
				**300**	0	846066	1614863
				**500**	0	2033997	4769469
				**1000**	966747	8018542	22978460

[1]Source: CORINE (Copernicus Land Monitoring Service) 2018, [2]Source: CBS (Central Bureau of Statistics Netherlands) 2017, [3]Source: NWB (National Road Network Netherlands) 2017.

Table S2: Coefficients of SLR

a. An overview of coefficients in the SLR model based on mobile measurements in Amsterdam for NO_2.

Variable	Estimate	StDev	Pvalue
Intercept	6.96	0.09	<0.001
Traffic intensity on major roads in a 50-m buffer	1.58*	0.05	<0.001
Length of major roads in a 100-m buffer	1.39	0.11	<0.001
Population density in a 5000-m buffer	2.13	0.06	<0.001
Traffic intensity on the nearest road	1.31	0.04	<0.001
Area of ports in a 1000-m buffer	0.58	0.04	<0.001
Traffic intensity on all roads in a 500-m buffer	2.00	0.07	<0.001
Length of major roads in a 50-m buffer	2.20	0.10	<0.001
Area of transportation services in a 5000-m buffer	1.29	0.06	<0.001
Area of ports in a 5000-m buffer	1.03	0.07	<0.001
$R^2 = 0.49$			

b. An overview of coefficients in the SLR model based on mobile measurements in Amsterdam for UFP.

Variable	Estimate	StDev	Pvalue
Intercept	11022.6	293.9	<0.001
Traffic intensity on major roads in a 50-m buffer	3915.2*	152.4	<0.001

Heavy-duty traffic intensity on nearest major road	1166.8	59.5	<0.001
Area of industry in a 500-m buffer	789.8	78.9	<0.001
Heavy-duty traffic intensity on major roads in a 100m buffer	1355.9	144.3	<0.001
Area of residential land in a 5000-m buffer	2871.7	162.9	<0.001
Area of industry in a 5000-m buffer	2689.2	167.9	<0.001
Area of transportation services in a 1000-m buffer	1539.7	109.1	<0.001
Area of water in a 300-m buffer	450.8	36.8	<0.001
Area of airport in a 5000-m buffer	560.5	49.0	<0.001
R^2 = 0.20			

Note: *Regression slopes and standard error (between brackets) are multiplied by the difference between the 10th and 90th percentile for all variables.*
